# Ten simple rules for thriving in a post-baccalaureate research program

**DOI:** 10.1371/journal.pcbi.1013174

**Published:** 2025-07-02

**Authors:** Michaelle E. DiMaggio-Potter, Angelica Velosa, John C. Brent IV, Osmar Del Rio, Eyerusalem F. Abebaw, Fernando Aguilar-Ortega, Dante Rogers, Patrick E. Rothwell, Angeline J. Dukes

**Affiliations:** 1 Department of Psychiatry and Behavioral Sciences, University of Minnesota, Minneapolis, Minnesota, United States of America; 2 Department of Neuroscience, University of Minnesota, Minneapolis, Minnesota, United States of America; 3 Department of Neuroscience, Baylor College of Medicine, Houston, Texas, United States of America; 4 Department of Pediatrics, University of Minnesota, Minneapolis, Minnesota, United States of America; Carnegie Mellon University, UNITED STATES OF AMERICA

## Introduction

Congratulations! You’re either starting your post-baccalaureate (post-bacc) journey, waiting on an acceptance letter, or considering this amazing opportunity for the future. Either way, you might be unsure how to make the most out of this experience. Well, you found the right place! At the time this article was written, the authors were nearing the end of their post-bacc journeys and eager to share their hard-earned wisdom on successfully navigating these programs.

As we begin, let’s clarify what post-bacc programs are. Generally, post-bacc experiences provide some combination of coursework, research experience, clinical opportunities, and/or professional development to students who have recently completed their bachelor’s degrees. However, the expectations and outcomes obtained vary depending on the program. The most common types of post-bacc programs are for students interested in applying to medical school or PhD programs. Pre-medical post-bacc programs often require you to pay for these experiences and offer classes or clinical opportunities to strengthen your qualifications. This can be helpful for students seeking to improve their GPA or gain clinical experience before applying to medical school.

**The focus of the present article is on research post-bacc programs that prepare recent college graduates (hereafter referred to as scholars) to pursue PhDs.** These research-focused experiences are typically fully funded and provide scholars with an annual salary as well as additional benefits such as relocation stipends, health insurance, and funding to attend conferences. There also may be opportunities to take courses. The bulk of your time is spent in a laboratory gaining hands-on, full-time research experience. These initiatives typically last 1 or 2 years and aim to help students become more competitive graduate school applicants [1]. The authors of this article are members of the first three cohorts of post-bacc scholars from the Minnesota Inclusive Neuroscience Development Scholars (MINDS) program, along with their program directors. The MINDS program is a fully funded, 2-year, post-bacc initiative that provides research opportunities and career development workshops to prepare scholars to pursue PhDs in neuroscience.

**Fig 1:**
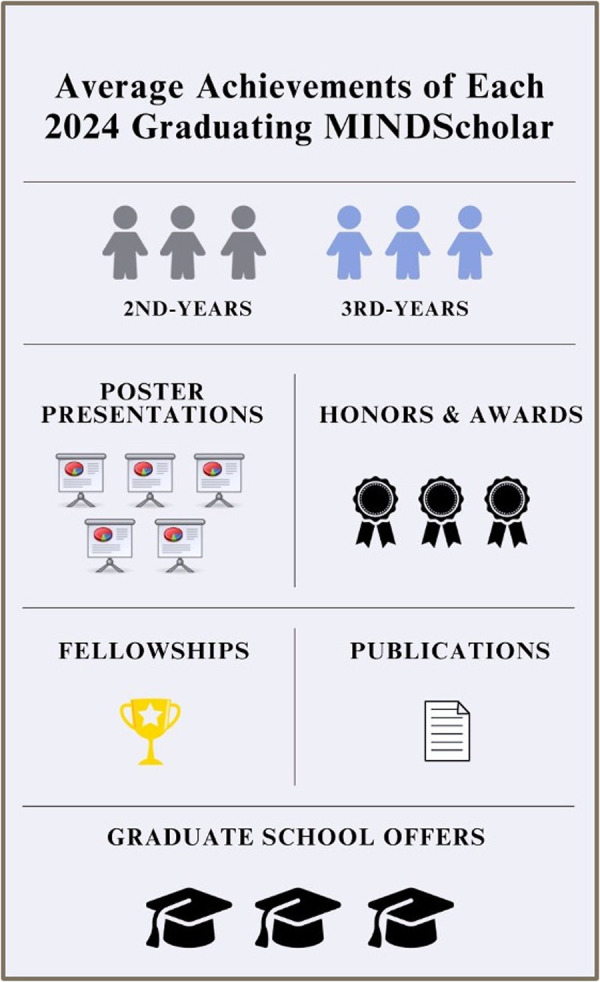
MINDScholar average achievements at program completion.

Post-bacc research programs can be helpful in preparing scholars for graduate school by enhancing both valuable technical skills and essential personal growth. For instance, several studies examined the efficacy of the National Institutes of Health’s Postbaccalaureate Research Education Programs (PREP), which span 1 year and aim to support students who seek enrollment in biomedical PhD programs. One study found that scholars’ GRE scores significantly improved after completing the program [2]. Moreover, scholars reported significantly increased confidence in conducting research, communicating scientific findings, directing research projects, understanding scientific literature, and earning acceptance into PhD programs [2,3]. Finally, 85% to 95% of PREP scholars transitioned directly into graduate programs, including biomedical PhDs [2–4]. These findings highlight the potential for post-bacc initiatives to broaden participation in biomedical PhD programs. Additionally, the MINDS program gathered data on the tangible benefits that scholars received after completing the program either at the end of their second year or if they extended their participation to a third year. These tangible outcomes included an average of five poster presentations at various conferences, co-authorship on one peer-reviewed publication, and multiple offers of acceptance to graduate programs (Fig 1). Intangible benefits of participating in a post-bacc program for the graduated MINDScholars included having the time and opportunity to explore academic interests, gaining confidence in pursuing a doctoral degree, and building a supportive community of peers with similar career goals. Participating in MINDS allowed all co-authors of this article to ultimately earn acceptance into various graduate programs of interest including clinical psychology, neuroscience, psychology, and even pharmacy school!

Figure 1 shows an infographic summary of achievements reported by the six MINDScholars who completed their post-bacc program in 2024. Each MINDScholar, on average, presented five posters, obtained three honors and awards, attained one fellowship for graduate school, published one peer-reviewed paper, and earned three graduate school offers of admission. Achievements were averaged among the six scholars and rounded to the nearest whole number. Three scholars completed the program in 2 years and three scholars completed an additional third year beyond the standard 2-year duration.

Now, we aim to leverage our experiences and perspectives to offer advice on how others can thrive in a research-focused, post-bacc program. Specifically, we define key aspects of becoming a successful post-bacc researcher, then offer strategies for enhancing personal and professional skills. By the end of this article, you will have gained insight into the realities and benefits of completing a post-bacc program as well as actionable steps you can take to thrive at this stage!

### Rule 1: Know what a post-bacc is (and isn’t)

If you are currently an undergraduate student seeking research experience, consider applying to undergraduate-focused research programs such as the National Science Foundation’s Research Experiences for Undergraduates, the Ronald E. McNair Scholars Program, or the Gates Millennium Scholars Program. On the other hand, if you have already earned a bachelor’s degree and are seeking more long-term research experiences, a post-bacc or master’s degree program may be a good fit. Master’s programs have more extensive coursework requirements in addition to research and do confer a degree upon completion, whereas post-bacc programs mainly focus on research, have fewer courses if any, and do not result in an additional degree. Master’s programs consider you a student and you pay tuition to attend. But most post-bacc programs consider you an employee and pay you a stipend or salary to participate. It is important to understand which program aligns with your goals.

Once you have determined if a post-bacc program aligns with your journey, you should learn the goals of your specific post-bacc program. Generally, post-bacc programs aim to strengthen scholars’ competitiveness as graduate applicants through providing mentored research experiences, professional development workshops, and a supportive community. This puts you in a unique position compared to other trainees in a lab because you have dedicated time and guidance for graduate school preparation.

To gain a better understanding of what niche post-bacc programs fill, let’s consider research technician (tech) positions, which are also commonly pursued after obtaining a bachelor’s degree. Research tech positions entail paid work that often involves maintaining the daily functions of a lab. Although a research tech’s responsibilities change depending on the lab, individuals in this position are typically not ensured ownership of a research project and do not have dedicated time or guidance to prepare for graduate or professional school. On the other hand, as a post-bacc scholar, your program is designed to offer opportunities for gaining independence in a focused research project, cultivating your skills as a scientist, and developing the personal/professional skills for you to thrive as a graduate student. In this role, you are essentially a PhD student in training working under the guidance of a Principal Investigator (PI), also known as the research faculty member who runs the lab, to gain scientific competence. Ultimately, you should be using this time to work on one (or more) projects, read literature, generate hypotheses, design and conduct experiments, analyze data, interpret results, and communicate your findings. Additionally, the post-bacc program should enhance the other skills necessary for becoming a well-rounded scientist, such as time management. Review your program’s mission statements or ask your director(s) about the program’s specific goals so you know what to expect.

### Rule 2: Manage your time

The transition from taking full-time coursework as an undergraduate student to conducting full-time research as a post-bacc scholar can be difficult. Many of us struggled with creating structure for each day. Fortunately, our struggles with structuring our time allowed us to identify different, useful tools for managing time effectively and developing healthy work habits to support this transition. We hope these tools can help you too.

First, reflect on how you’ve worked best in the past, whether that was completing assignments during your undergraduate studies or while engaging in your favorite hobbies. Here are some questions to get you started:

- Are you more productive working by yourself or closely with others?
- Do you work better in person (e.g., in the lab space) or remotely (e.g., at home)?
- Are you more motivated to complete tasks in the morning or evening?
- Can you work in loud environments or do you need quiet ones?
- Do you work more effectively when your schedule is flexible and spontaneous or structured and consistent?

Now, use these reflections to mold your work environment to fit your needs! For instance, if you know you work best with consistency, in the morning, and with others, try scheduling regular morning meetings with lab mates or friends who also work best this way. You can each work on your own individual projects, but the presence of others will keep everyone accountable to stay on task. By doing this, you will likely focus better and work more efficiently. This can be particularly effective when working on graduate school applications to make steady progress.

After settling in the lab, explore online or physical calendar systems to visualize your time commitments and deadlines. Consistently using calendars has helped our scholars identify how they’re spending their time, create structure for each day, determine if they have time for new projects, and adjust future plans according to the actual amount of time spent completing each task. In laboratory settings, there will be projects without preset deadlines that require consistent, independent motivation. For projects like these (e.g., reading literature or building new coding skills), try creating specific deadlines with your mentor for accountability.

Even when you’ve blocked out chunks of time to work on your calendar, you may find it challenging to follow through; perhaps, you feel unmotivated to start a project or find it impossible to restart a task once distracted. We often find ourselves pacing around our lab or living space, actively avoiding writing a research paper or personal statement. For these moments, we recommend setting a 15-minute timer and giving yourself the opportunity to work on the task with no expectations in terms of quality. The goal is to simply write something down. After the timer is done, check in with yourself to see how you are doing. More often than not, starting the task builds the motivation to keep going and reduces feelings of overwhelm. A task can seem overwhelming because we build up in our minds that it’s very difficult or it needs to be perfect after the first attempt. This strategy helps overcome the initial hurdle of starting. Furthermore, restarting a task when your work rhythm is interrupted can also be a challenge. In this case, implement time management methods that are curated to improve concentration and limit mental fatigue. One example is the Pomodoro method [5], a time management system that splits long work sessions into 25-minute working blocks with 5-minute breaks in between. This method allows you to focus intensely on your work, take a brief break, and then jump back into the task more readily. During breaks, avoid activities that encourage prolonged distraction, such as scrolling through social media. Instead, engage in energizing activities such as stretching, meditating, eating a healthy snack, or taking a quick walk. These time management techniques can help you get started and keep powering through your to-do list.

If you need extra guidance in identifying other tools, ask your peers in the lab about their favorite time management strategies. Lab managers, research techs, other post-baccs, and graduate students who more recently transitioned into full-time scientific research can provide valuable advice. Leverage their experiences to identify new habits to pursue and which to avoid.

Although we mentioned specific time-management strategies, the best approach will be unique to you. Try some of our suggestions, discuss various tactics with peers, and search online for more resources. Remember that learning new skills is challenging. It requires self-compassion and multiple attempts. However, know that you are capable of discovering effective time management strategies that will help you excel in this new environment.

### Rule 3: Practice your craft

The bulk of your time in a research-focused, post-bacc experience will be spent, of course, conducting research. If you have not previously had opportunities to conduct research full-time, it usually involves working on a research project and gaining greater independence in executing it. This process requires the acquisition of knowledge, skills, and habits that will allow you to practice the craft of research as a post-bacc and beyond.

(1) *Knowledge*. One of the first tasks in your research lab will be to immerse yourself in the current state of knowledge related to your project by reading relevant papers (especially those previously published by your research mentor/lab). Most research projects are motivated by specific gaps in knowledge, but identifying and defining these gaps is really difficult—even for experienced researchers! Have conversations with your mentors and lab mates about why a particular topic has been chosen. Listen to conversations in lab meetings and seminars, ask questions about things you don’t understand, and get recommendations for additional articles to read. Remember that it takes a long time and consistent effort to become an expert. Having reasonable expectations of what you’ll learn as a post-bacc scholar is imperative to help dissuade feelings of imposter syndrome. Imposter syndrome includes feelings of inadequacy and self-doubt in your capabilities, skills, and accomplishments, despite evidence of your past success [6]. While you won’t become an expert in the field during your post-bacc program, you can begin to develop a foundation of knowledge to carry with you in graduate school. You’ll be surprised by how much you can learn by the end of the program through asking questions, reading articles, and engaging in discussions!

(2) *Skills*. You also need to be trained and develop proficiency with the hands-on methods required to conduct your research project. Other members of your lab may be responsible for teaching you these skills at the beginning of a project, though sometimes you may take the lead on developing a new method. In either case, problems often arise that require troubleshooting. Don’t be afraid to talk through these challenges with your lab mates and PI—they’re there to help!

In addition to collecting data, the ability to think about data is also an essential skill for any researcher. This includes interpreting what raw data means as they are being collected, as well as visualizing and statistically analyzing complete datasets into graphs and figures. Your own research project should provide opportunities to practice your craft in these areas, but you can also develop these skills by thinking about the data that other people present in lab meetings or seminars. Practice interpreting their data yourself and questioning if it could be analyzed differently. You may provide a new perspective that they hadn’t considered! Moreover, enhancing your science communication skills is another essential area of growth during your post-bacc experience. All scientists have to learn how to effectively communicate their research findings to a wide variety of audiences, ranging from other researchers to the local community. Be active in practicing multiple forms of communication from giving poster and oral presentations at conferences to creating written documents (abstracts/manuscripts/grants) and engaging with the public through outreach at K-12 schools or community events.

(3) *Habits*. Your post-bacc research experience is also a great opportunity to develop productive habits that can set you up for a successful scientific career. One great example is time management, as described in the previous rule. Another useful habit to practice is pushing your own boundaries to try new things that feel challenging, whether it’s a new laboratory technique or raising your hand to ask a question during a research seminar. Practice good record-keeping habits by keeping a detailed lab notebook with information about your experiments, developing data storage systems that make it easy to find things in the future, and using calendar systems to keep track of activities inside and outside of the lab. Finally, curiosity is an important trait for practicing scientists, which can be nurtured through habits like verbally asking questions or searching for answers to questions by conducting literature searches on PubMed or Google Scholar.

Through acquiring new knowledge, developing your technical skills, and forming solid habits, you can begin to hone your craft as a post-baccalaureate researcher.

### Rule 4: Uncover the hidden curriculum

As you expand your research skills and knowledge, you may realize that academia also requires learning the hidden curriculum. The “hidden curriculum” refers to the unspoken rules, expectations, and practices within research and academia [7,8]. It includes aspects such as how to properly construct a scientific poster, dress for conferences, advocate for your research ideas with faculty, network with potential collaborators, seek additional mentorship, and create an effective CV. All of these are important aspects of being a researcher, yet they’re often not explicitly taught. This may lead you to assume that you should inherently know how to do them, but this is not the case. Much of the “how” in academia can be mysterious and involve long processes of trial and error. Beginning to tackle the hidden curriculum as a post-bacc scholar can save you a lot of time in the future and set you up for success.

Navigating the hidden curriculum can feel like playing an elaborate game without knowing the rules, especially if you don’t know resources exist. Previous Ten Simple Rules (TSR) papers such as “TSR for reading a scientific paper,” “TSR for making good oral presentations,” or “TSR for good poster presentations” [9–11] and other skill development-focused articles, such as *A Quick Guide to Networking for Scientists* [12] can be immensely useful to read. Jessica Calarco’s *A Field Guide to Grad School* [13] is another great resource and offers a comprehensive guide for navigating research as a trainee. Although it is primarily directed toward PhD students, post-bacc scholars may also find relevant and helpful advice on how to thrive in academia. In addition to these resources, openly ask for support from your post-bacc mentors and fellow peers. It can be hard to admit when we’re unsure how to do something, but other people won’t realize that you need help figuring it out unless you vocalize it. For example, you can engage in peer review sessions to get feedback on your science writing, CV, or poster. You can also practice giving presentations during your lab meetings. You can even directly ask people in your lab what they wish they knew at your stage, how they navigated their first conference, and/or found mentors. Immersing yourself in these practices will not only highlight *how* to do these activities but will also bolster your comfort and confidence in asking for guidance in future tasks that you’re uncertain of.

Uncovering the hidden curriculum takes patience and practice, but it also requires showing yourself grace. Placing high, unrealistic expectations on yourself or comparing yourself to others won’t accelerate the process. In fact, doing so can exacerbate feelings of isolation and imposter syndrome, especially if you come from an underrepresented background or are a first-generation college student. Trust that you’re meant to be where you are, and use the resources at your disposal to uncover the hidden curriculum.

### Rule 5: Bounce back from setbacks

As you navigate the hidden curriculum and progress through your post-bacc experience, you will inevitably encounter setbacks. Whether it’s a failed experiment, a rejected application, or constructive criticism on a presentation, these situations may be disheartening and impact your self-esteem. Here are some ways the MINDScholars have successfully dealt with setbacks:

*Embrace a growth mindset* [14]. If an experiment goes wrong, consult with colleagues and mentors who might offer new perspectives, re-evaluate your methods, and try again. Sometimes instead of pushing through and re-attempting a task the same day, stepping away and returning to it the next day can provide a fresh perspective and a clearer approach to the problem. Persistence is key. Furthermore, maintaining a growth mindset is essential when receiving constructive criticism. While it is challenging to avoid viewing negative feedback as a personal attack, remember that the people providing feedback want you to succeed. Think of the critiques as opportunities for growth. Constructive criticism can highlight areas for improvement that you might have overlooked. Use this feedback to refine your work and develop your skills further. If you have trouble receiving criticism in real-time, you could ask for written comments to read and process later. Additionally, be sure to keep track of any positive feedback you receive—having a record of your strengths can serve as a valuable reminder of where you excel and can boost your confidence.

#### Seek support.

Developing a solid support system is essential. Surround yourself with people who can offer encouragement, advice, and empathy. Friends, family, and mentors can provide invaluable support during challenging times; but the caveat is that you have to be willing to tell them when you’re struggling for them to provide sufficient help. We also recommend finding a professional therapist, even if you don’t feel you need one right now. Having a continual therapeutic relationship with a dedicated professional can offer preventive care when things are going well, and help you navigate stress and pressure when life becomes harder.

#### Value the process.

Applying for travel awards, research funding, and graduate school can be both confusing and competitive. Fellowships, in particular, are highly competitive, and it is common not to succeed on the first attempt. However, the application process itself is valuable; as you refine personal statements and gather constructive feedback from reviewers, this can strengthen your future applications. We want to emphasize that if you apply and get rejected from graduate school, it is not the end of the world. The competition for graduate school positions is intense, and facing rejections is a very common experience. These rejections are not a reflection of your worth or capabilities. Often, it’s a matter of fit and the availability of positions. A prime example is our third-year MINDScholars who did not get accepted into their desired graduate programs when they first applied. Instead of giving up, they remained committed to their goals and made the most out of the additional year as post-bacc scholars to further develop their independent research skills. Specifically, they focused on acquiring new technical skills, attaining authorship on manuscripts, and presenting at more conferences to grow their professional networks. All of these pursuits were instrumental in helping them gain offers of acceptance into their desired graduate programs when they applied again.

If there is one key takeaway from this rule, it is to be kind to yourself. Celebrate the small victories and acknowledge your progress, no matter how minor they may seem. During your time as a post-bacc scholar, you will make mistakes, learn from them, and build the foundation necessary to succeed in graduate school. Setbacks can be disheartening, but they are valuable opportunities to learn new skills, improve your abilities, and develop the resilience needed to overcome future obstacles.

### Rule 6: Learn to be your best advocate

In addition to recovering from setbacks, developing strong self-advocacy skills is vital during your post-bacc. Self-advocacy involves identifying and communicating your needs to others. Doing so effectively allows you to tailor your post-bacc experience and propel you toward achieving your short- and long-term goals.

To improve your ability to self-advocate, start by identifying your long-term goals. Then, determine any gaps you may have that could impede your ability to fulfill these aspirations. If you are unsure what those gaps might be, seek out professionals who are doing your dream career. Ask them about their journeys as well as advice on what they wish they knew at your stage. Finally, set clear milestones to help you progress toward your objectives. If you do not know the appropriate steps to take, that’s okay! This is actually a great time to self-advocate. Request a meeting with your PI or program director(s) and discuss the activities that generally interest you (like teaching or research) to learn about potential career paths. Ask what knowledge and skills are imperative to reach your goals and then use this information to determine what you need to learn. Once you’ve identified areas you need support in, express them to your PI and program directors. In Table 1, we’ve provided example inquiries and requests. This list is by no means exhaustive; if you have other needs, bring them up with your mentors!

**Table 1:** Example inquiries for self-advocacy.

Self-Advocacy Category	Example Inquiries and Requests
Professional development	• What steps do I need to take to initiate an independent project? • How do I effectively manage my time and prioritize different responsibilities?
Research	• I’m really interested in learning this technique. Can I join this research project in the lab? • I don’t fully understand [this topic]. What articles should I read or other resources do you recommend for me to gain a better understanding?
Funding opportunities	• Do you have suggestions for departmental funds or travel awards that I could apply for?
Endorsement	• Would you be willing to sponsor me for a conference membership? • Would you be able to write me a strong letter of recommendation for this fellowship?
Document processing	• Could you please edit and approve of my poster?
Networking	• I’d like to use this data collection tool/discuss navigating academia with my identities/collaborate with this principal investigator (PI)/etc. Would you be willing to connect us?
Work enhancements	• It would really help my research to obtain a laptop/other work equipment through the lab. • I would love to meet more regularly with you/increase the length of our meetings.
Personal development	• I am experiencing a personal challenge (e.g., one related to my identity, financial situation, etc.). Could you provide guidance on how to navigate this? How might you have overcome similar challenges?
Feedback for program directors or PI	• I think [this element of the program] could be improved. • I have [this concern] about our lab’s interpersonal dynamics.

When asking for help, you may feel like a burden on others. Or perhaps, you think your PI might become secretly upset with you for taking too much of their time. Or maybe you believe you should already know the answers to your problems and educate yourself alone. These thoughts are understandable and common among trainees. However, many mentors genuinely want to help guide you. Thus, you are not expected to know everything and should not have to figure it out alone. You can work with your PI to build trust over time. As you build a supportive space with them, it may become easier to ask for support. Additionally, it is the responsibility of your mentors to inform you of their limitations and enlist others to provide additional guidance whenever necessary. Although we encourage transparency with your PI, we also recognize that there is an imbalance of power in these relationships. Unfortunately, there are situations in academia where students experience discrimination including racism, classism, heteronormativity, and sexism, among other forms of unfair treatment. If you feel unsupported by your mentor, consider practicing another form of self-advocacy by reaching out to a different mentor, such as a program director or another faculty member, to discuss how to get your needs met and how to navigate your relationship with your primary PI. Overall, we want to highlight that you are not a burden, and you have every right to receive support.

Lastly, unforeseen circumstances, such as mental health concerns or family emergencies, may become obstacles in your post-bacc growth. You are not the first trainee to experience them and don’t have to navigate them on your own. Be brave in sharing these difficulties with your PI. Discuss possible solutions, such as deadline extensions, removal from a project, or time off*.* Requesting these accommodations can be very challenging because you may feel pressured to maintain a consistently high work ethic in academic settings. If you sense yourself feeling overwhelmed or falling behind, try to resist the urge to avoid or gloss over details in meetings and, instead, approach the topic, describe the challenges, and ask for help. Though you may feel uncomfortable, your PI will likely appreciate the transparency. You will also be more effective at completing your remaining tasks and feel more empowered to assert your needs, both of which will support your success now and in your future career.

### Rule 7: Cultivate your community

As you advocate for yourself in pursuit of your personal and professional goals, you may realize that you need additional support to propel you forward. Cultivating a supportive community during your post-bacc program will greatly enhance your well-being and success. Oftentimes when we think of our support system, we identify family, friends, and peers as emotional support networks. This group of people, albeit indispensable, cannot support you in all aspects of your career. You will need to establish professional relationships that offer support in various domains including research, career guidance, and advocating on your behalf to others.

Building your community—or in other words, networking—can be anxiety-inducing and stressful. However, it may feel more manageable when you realize that you’ve already begun to develop a network simply by participating in a post-bacc program! First and foremost, develop meaningful connections with fellow post-bacc scholars. Your peers, having experienced the program alongside you, can offer a level of understanding and perspective that no one else can. As everyone moves beyond the post-bacc stage, maintain these relationships by sharing information about research positions or professional development programs, advocating on each other’s behalf for future jobs, or simply commiserating together about the trials of graduate school. Next, forge connections with members of your lab (e.g., PI, lab staff, graduate students, and postdoctoral researchers) as well as other faculty and graduate students in the department. These researchers will help you hone your technical skills, provide strong scientific feedback, give experimental guidance, offer honest insight into the lab-specific culture, and support you as you enter this new space. Pro tip: to maintain these important relationships after you finish your post-bacc program, email annual updates to your former PI and lab mates!

Once you have begun cultivating a community within your own institution, broaden your network to include the greater scientific community. A great way is through networking at conferences. Prior to attending a conference, review the conference program and identify poster abstracts and sessions that interest you. If you are interested in someone’s research, email them before the conference to arrange a meeting over coffee or chat with them after their presentation. Discuss mutual scientific interests, their career trajectory, or ask for general advice as a research trainee. If this idea makes you uneasy, ask your PI or senior lab members to introduce you to conference attendees that they know. Another great way to expand your network is through the use of social media [15]. There are several platforms where scientific communities thrive, including X (formerly Twitter), Bluesky, LinkedIn, and even TikTok. These are great resources to explore new research and showcase your accomplishments. Returning to a previous example, try connecting with scientists you meet at conferences by connecting with them on LinkedIn or following them on social media to maintain the connection. You can also join established organizations to connect with other scientists of similar identities such as Black In Neuro, Cientifico Latino, American Indian Science and Engineering Society, or Out in STEM (oSTEM). Last, but certainly not least, look to connect with your local non-scientific community. With so much of our time dedicated to science, we often overlook the communities we hope to serve as researchers. Connecting with local schools or organizations is a fantastic way to engage with the broader community, disseminate your research knowledge, and build lasting relationships.

**Fig 2:**
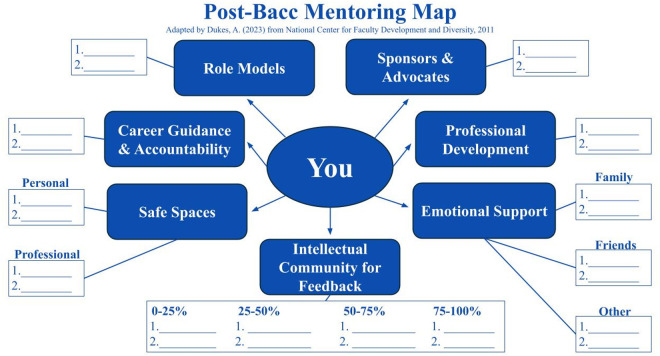
Post-bacc mentoring map. A mentoring map (adapted by Dr. Dukes in 2023 from the National Center for Faculty Development and Diversity Mentoring Map, 2011) for post-baccalaureates to fill in to identify areas of support and potential growth.

The network you shape for yourself will play a crucial role in both your personal and professional development. Prioritize building a scientific community that you feel a part of, confident in, and supported by. To determine what your current community looks like, we recommend filling out the post-bacc mentoring map (Fig 2). This map was adapted for post-bacc scholars by Dr. Angeline Dukes in 2023 and is inspired by the National Center for Faculty Development and Diversity Mentoring Map [16]. Please note that at this stage of your career, one person will likely serve in multiple capacities in your mentoring network; this is to be expected. Also, do not feel discouraged if you cannot completely fill out the entire map. This is a tool meant to help you identify areas where you can network and grow your professional community to obtain additional mentorship. Investing time and effort into establishing and maintaining these relationships will foster mutual support and scientific growth during your post-bacc experience and beyond.

**Role Models**—Individuals you can look up to as examples of success. This can include people who have achieved your desired goals and/or have character and values that you want to embody.

**Sponsors and Advocates**—Influential individuals (e.g., faculty, program directors, and administrators) who are present in spaces you would not have access to as a trainee and can speak highly of you.

**Career Guidance and Accountability**—Mentors who currently or previously work in your desired career and can help hold you accountable for making progress towards your career goals. They can also connect you with people from the networks and opportunities that you may not be aware of.

**Professional Development**—People, on-campus resources, or programs that can enhance your development or share access to opportunities for you to grow your skillset.

**Safe Spaces**—Mentors, colleagues, and friends you can speak to openly without fear of judgment or repercussions. We recommend finding safe space people in both personal and professional settings.

**Emotional Support**—Trusted individuals you can turn to for support during difficult times.

**Intellectual Community**—People who can offer feedback at different stages of a scientific product (e.g., creating a research poster or writing a review paper).

- 0%–25% → Initial brainstorming and rough draft stages of a project
- 25%–50% → Completed first draft
- 50%–75% → Revised drafts
- 75%–100% → Final details before product submission

### Rule 8: Discover your passions outside of the lab

Your community can also help you discover passions beyond research. While the lab is where you’ll spend the bulk of your time as a post-bacc, it isn’t everything. You should explore other aspects of your future career because engaging in passions outside of research is a great way to build professional and personal relationships, enhance self-care, and develop career-relevant skills [17,18]. Additional interests may include teaching, mentorship, community engagement, and volunteering.

To begin your journey beyond the lab, tap into your program’s and institution’s resources. This can include connecting with a teaching or outreach center, earning certificates via self-paced online courses, or attending relevant workshops. Gain mentorship experience by advising undergraduate research assistants in your lab through their scientific projects. Additionally, obtain teaching experiences by engaging with your community. Some examples include tutoring local high school students, giving a guest lecture at your former undergraduate institution, or conducting experiments at science booths during community festivals. Immerse yourself further in your local community and strive to volunteer with existing organizations. This could be with local nonprofits and community service projects involving at-risk youth, individuals experiencing homelessness, prisoners, environmental cleanup, or any other cause that inspires you. We highly recommend exploring opportunities to teach, mentor, and volunteer during your post-bacc to discover your passions and demonstrate that you can actively participate in these pursuits while conducting full-time research. Delving into such hobbies also supports your community’s growth and builds essential skills for your future career.

Beyond the aforementioned activities, we encourage you to push past your comfort zone and involve yourself in extracurriculars that are unrelated to your career. This might include creative arts, such as painting, composing music, or writing poetry. It may also pertain to physical activity like playing a sport in a recreational league or participating in your university’s wellness program. Perhaps, you like technology-related hobbies and would enjoy coding through publicly available online resources or tinkering with electronics. Whatever your interests are, nurturing your passions outside of the lab and learning to balance them with your scholarly pursuits will profoundly impact your productivity and overall well-being in academia.

### Rule 9: Care for yourself

We may be post-bacc scholars, researchers, and scientists—but we are humans first and foremost. Your well-being must be a priority. While your accolades and research experience are instrumental for advancing your career, they are not everything. Unfortunately, it is not uncommon for scientists to let our dedication to research take a toll on our mental health. Everyday stressors that PhD students encounter—such as poor work–life balance, imposter syndrome, the hypercompetitive culture of research, and toxic productivity [19,20] —can also negatively impact your health and well-being as a post-bacc. To navigate these stressors and the toxic atmosphere they can foster, we urge you to develop self-care strategies to protect your well-being.

Self-care is misrepresented and dramatized in the media as time-consuming and expensive self-pampering. While spa facials and massages are a form of self-care, more consistently attainable examples include taking a long shower, cooking a comforting meal, getting enough sleep, exercising, and spending leisure time outdoors. Your version of self-care is unique and can change over time. It’s a lifelong process that adapts to your current needs and interests. Although it looks different for everyone, self-care at its core entails taking a break. Working too many hours in one sitting is counterproductive and can be detrimental to your mental and physical health. Merely checking off additional tasks will often exacerbate stress rather than relieve it, leading to burnout—a state of emotional, mental, and often physical exhaustion that emerges from chronic, work-related or interpersonal stressors [21].

You can prevent burnout by using strategies mentioned in previous rules: community support, time management, and self-advocacy. We emphasize that *you are not alone* and advise you to lean on the support systems you’ve built. Openly share when you’re struggling; when those moments of self-doubt, frustration, and imposter syndrome creep in. We know that this requires a good level of emotional vulnerability, but it opens the conversation up for peers who are silently suffering and enables others to share advice on how they navigated similar challenges. You can also ask others how they prioritize self-care to get new ideas for yourself! Moreover, burnout prevention can occur through self-advocacy in the form of boundary-setting with mentors and colleagues. Regardless of how rewarding your responsibilities may be, they will require time and energy. Using time management techniques and maintaining firm boundaries allows you to dedicate enough time to these commitments and your well-being. While setting boundaries can be a difficult practice, it is a necessary skill best learned at the beginning of your career. Boundary-setting combines self-advocacy and self-care; by recognizing and communicating your needs, you set clear expectations and foster an environment where you can thrive. Practicing consistent self-care routines and prioritizing your well-being will allow you to continue doing what you love and loving what you do for many years to come.

### Rule 10: Enjoy it!

Each of these rules highlight how to thrive in a post-bacc research program, but it is equally important for you to take the time to truly enjoy the experience!

Developing and maintaining a sense of enjoyment is beneficial for both navigating challenges and maximizing the benefits of being a post-bacc. A positive and engaged mindset enhances motivation, deepens commitment to research, and ultimately improves productivity [22]. Enjoyment also plays a role in information retention, fosters curiosity, and encourages the development of novel research ideas. When you are passionate and excited about your work, that positivity can be contagious to others which makes it easier to build a strong professional network. But please note, making genuine connections with others also requires the honest vulnerability, that we mentioned earlier, when things are not going so well. Having this support system during low moments makes it even more meaningful to celebrate the wins with them!

Although finding and sustaining joy throughout this experience can be challenging, there are proactive steps that you can take. A great way to start is by engaging in mental self-care and reframing the barriers you face. You can challenge negative thoughts by questioning if they are grounded in fact—which they are often not—and identifying actionable steps to take. For instance, challenge the thought “I’m not smart enough to become an expert in this subject” by remembering how much you learned in college and grounding yourself in the fact that every expert was once a beginner. Then create a plan of reading one paper a week to increase your knowledge. These efforts to reframe negative thoughts and replace them with more balanced perspectives can help boost your self-confidence and make obstacles seem easier to overcome.

In summary, although you will face personal and professional obstacles during your post-bacc program, try to challenge negative thoughts and implement strategies that promote enjoyment. Embracing this approach will help you thrive and make the most out of this unique experience. Overall, your post-bacc journey is a wonderful time for you to make new friends, build your confidence as a researcher, and discover your passions. Have fun, believe in yourself, and implement the skills and knowledge we’ve discussed to pave your *own* path to success. We genuinely hope that you enjoy this opportunity as much as we have!

## Conclusion

This TSR article outlined tips for scholars to thrive in a post-bacc research program, such as how to self-advocate and establish supportive communities. We recognize that in research, there is always work to be done and more questions to be answered. Yet, learning to set boundaries and manage your time to engage in self-care are useful habits to pick up early. Give yourself grace; remember that you are a trainee, will inevitably make mistakes, and will definitely grow from them. When you struggle, remind yourself that you were carefully selected for every opportunity you attain, and it’s okay to lean on the supportive networks you built. Most importantly, never forget that you are here because you enjoy what you do and are more than capable of succeeding.

As you progress through your post-bacc program and begin applying to graduate school, other TSR papers can be helpful, as they focus on guiding prospective and current graduate students [23,24]. With our rules and these additional resources at your disposal, we hope you feel better prepared to successfully navigate a post-bacc program and flourish as a scholar. We can’t wait to see you thrive!
